# Multiple NUCLEAR FACTOR Y Transcription Factors Respond to Abiotic Stress in *Brassica napus* L

**DOI:** 10.1371/journal.pone.0111354

**Published:** 2014-10-30

**Authors:** Li Xu, Zhongyuan Lin, Qing Tao, Mingxiang Liang, Gengmao Zhao, Xiangzhen Yin, Ruixin Fu

**Affiliations:** 1 College of Resources and Environmental Sciences, Nanjing Agricultural University, Nanjing, China; 2 Jiangsu Key Lab of Marine Biology, Nanjing, China; National Taiwan University, Taiwan

## Abstract

Members of the plant NUCLEAR FACTOR Y (NF-Y) family are composed of the NF-YA, NF-YB, and NF-YC subunits. In *Brassica napus* (canola), each of these subunits forms a multimember subfamily. Plant NF-Ys were reported to be involved in several abiotic stresses. In this study, we demonstrated that multiple members of thirty three *BnNF-Y*s responded rapidly to salinity, drought, or ABA treatments. Transcripts of five *BnNF-YA*s, seven *BnNF-YB*s, and two *BnNF-YC*s were up-regulated by salinity stress, whereas the expression of thirteen *BnNF-YA*s, ten *BnNF-YB*s, and four *BnNF-YC*s were induced by drought stress. Under NaCl treatments, the expression of one *BnNF-YA10* and four *NF-YB*s (BnNF-YB3, BnNF-YB7, BnNF-YB10, and BnNF-YB14) were greatly increased. Under PEG treatments, the expression levels of four *NF-YA*s (BnNF-YA9, BnNF-YA10, BnNF-YA11, and BnNF-YA12) and five *NF-YB*s (BnNF-YB1, BnNF-YB8, BnNF-YB10, BnNF-YB13, and BnNF-YB14) were greatly induced. The expression profiles of 20 of the 27 salinity- or drought-induced *BnNF-Y*s were also affected by ABA treatment. The expression levels of six *NF-YA*s (BnNF-YA1, BnNF-YA7, BnNF-YA8, BnNF-YA9, BnNF-YA10, and BnNF-YA12*)* and seven *BnNF-YB* members (BnNF-YB2, BnNF-YB3, BnNF-YB7, BnNF-YB10, BnNF-YB11, BnNF-YB13, and BnNF-YB14) and two *NF-YC* members (BnNF-YC2 and BnNF-YC3) were greatly up-regulated by ABA treatments. Only a few *BnNF-Y*s were inhibited by the above three treatments. Several NF-Y subfamily members exhibited collinear expression patterns. The promoters of all stress-responsive *BnNF-Y*s harbored at least two types of stress-related *cis*-elements, such as ABRE, DRE, MYB, or MYC. The *cis*-element organization of *BnNF-Ys* was similar to that of *Arabidopsis thaliana,* and the promoter regions exhibited higher levels of nucleotide sequence identity with *Brassica rapa* than with *Brassica oleracea*. This work represents an entry point for investigating the roles of canola NF-Y proteins during abiotic stress responses and provides insight into the genetic evolution of *Brassica* NF-Ys.

## Introduction

Abiotic stress, such as salinity, drought or dramatic temperature change, adversely affects plant growth and productivity. Plants, being sessile, have evolved a range of strategies to adapt to detrimental environmental conditions. Plants respond to unfavorable conditions through two pathways, i.e., the abscisic acid (ABA)-dependent and ABA-independent pathway [1]–[3]. Transcription factors (TFs) play vital roles in mediating stress tolerance in both of these pathways.

NUCLEAR FACTOR Y (NF-Y) TFs are ubiquitous in higher eukaryotes and belong to the CCAAT-binding factor (CBF) family, also known as the Heme Activator Protein (HAP) family in yeast. The NF-Y heterocomplex consists of at least three subunits, namely NF-YA (also named CBF-B or HAP2), NF-YB (CBF-A or HAP3), and NF-YC (CBF-C or HAP5) [4]. In planta, each NF-Y subunit is encoded by a multi-member family, and there are at least 10 annotated members in each NF-Y subfamily in *Arabidopsis* [5]–[7]. Rice harbors at least 10 *NF-YA* genes, 12 *NF-YB* genes, and 8 *NF-YC* genes [8]. In a previous study, we identified 14 *NF-YA* genes, 14 *NF-YB* genes, and 5 *NF-YC* genes in *Brassica napus* L. [9]. NF-Y family members are extensively involved in plant development and stress responses, as summarized in a recent review [10].

Increasing evidence suggests that NF-Y subunits are important regulators of abiotic stress responses [11]–[14]. Overexpression of *NF-YA1* in *Arabidopsis* was shown to result in hypersensitivity to salt stress during early postgerminative growth [15]. Real-time PCR analysis revealed that *Glycine max* (soybean) *GmNF-YA3* was induced by ABA and other abiotic stresses, and that overexpression of *GmNF-YA3* in *Arabidopsis* enhanced drought resistance [16]. A microarray analysis identified *Arabidopsis NF-YB2* as one of the most strongly induced genes under multiple treatments, including cold, mannitol (dehydration stress), and NaCl (salinity) [17]. Further analysis confirmed that AtNF-YB2 functions in early flowering under osmotic stress [12]. AtNF-YB1 was found to improve plant performance under drought conditions and its ortholog in maize, ZmNF-YB2, was shown to resist drought stress in the field [11]. Several drought-related *NF-YB* genes were identified in *Triticum aestivum* (wheat), *Populus euphratica* (poplar), and *Hordeum vulgare* (barley) [18]–[20]. In the NF-YC subfamily, the transcript level of *AtNF-YC2* was highly induced by light, oxidative, heat, cold, and drought stress [21]. Overexpression of *PwHAP5* (homolog of *Arabidopsis NF-YC2*) from the conifer (*Picea wilsonii)* partially rescued the increased sensitivity of *nf-yc2* to salt, drought, and ABA treatments [22].

In addition, several members of the NF-Y family were shown to be regulated by the microRNA169 (miR169) family, suggesting that a complex regulatory cascade is activated under stress conditions [13], [14], [23]. Li et al. (2008) demonstrated that *AtNF-YA5* is a target of miRNA169a or miRNA169c [13]. At least six other miR169 targets were predicted to exist in the AtNF-YA family [24]. Of those six NF-YAs, NF-YA2, 3, 7, and 10 were found to be involved in multiple abiotic stresses, especially those involving low nutrient availability [25]. In rice, an *NF-YA* gene (*Os03g29760*) known to be a target of the miR169 family was found to be induced by high salinity [14]. The above results suggest that plant NF-Ys play a crucial role in the plant’s response to abiotic stress. However, the roles of BnNF-Ys under stress have not been examined in detail.

Canola (*B. napus*) constitutes the third largest vegetable oil source in the world. However, salinity and drought stress have a major limiting impact on its production and yield stability. Drought accounts for at least a 30% yield loss in canola every year [26]. In this study, we examined the expression patterns of *BnNF-Y*s under drought, high salinity, and ABA conditions. Our results suggest that 27 of 33 putative *BnNF-Y*s were induced by salt or drought stresses. Furthermore, 20 *BnNF-Y*s may be involved in the ABA-related pathway. We cloned the promoters of 28 *BnNF-Ys* from canola, which mostly included regions ∼1000****bp upstream of the coding sequences, and identified their stress-related *cis*-elements. Taken together, our results suggest that most *BnNF-Y* members are regulated by abiotic stresses in an abscisic acid (ABA)-dependent or ABA-independent manner and serve as an entry point to investigate the roles of these genes in the plant’s response to environmental stresses.

## Materials and Methods

### Plant materials and growth conditions

Seeds of oilseed rape (*Brassica napus* ‘Nanyanyou 1’, kindly provided by Professor Zhaopu Liu at Nanjing Agricultural University) were kept in darkness for 2 days and germinated in Hoagland nutrient solution. The plants were grown for 3 weeks in the greenhouse with a light intensity of 392∼415****µmol m^−2^ ****s^−1^ during a daily cycle consisting of 16****h of light at 25°C and 8****h of darkness at 18°C. For dehydration and salt stress treatments, plants were grown hydroponically in solution containing 15% (w/v) PEG6000 and 150****mM NaCl plus ½ Hoagland nutrient solutions for 1 or 3****h, respectively. For ABA treatments, 100****µM ABA was added to the ½ Hoagland nutrient solution and the plants were treated for 1 or 3****h. Seedlings growing on ½ Hoagland nutrient solution for various periods of time were used as the control. The harvested samples were immediately frozen in liquid nitrogen and stored at −80°C until used for gene expression analysis. All experiments were performed in biological triplicate.

### Genomic DNA extraction, total RNA isolation, and primary cDNA synthesis

Genomic DNA was extracted from oilseed rape using a method from Rogers and Bendich’s CTAB-based protocol [27]. Total RNA of each sample was extracted using an E.Z.N.A. Plant RNA Kit (Omega Biotek, Cat#R6827, USA). The quality and quantity of extracted DNA and RNA from all samples were confirmed by both agarose gel electrophoresis and spectrophotometry (Thermo Scientific, NanoDropTM 1000, USA). Total RNA samples were pretreated with an RNase-Free DNase Set (Omega Biotek, Cat#E1091, USA) to eliminate residual genomic DNA. Primary cDNA was synthesized using a PrimeScript RT-PCR Kit (TaKaRa Code: D6110A, Japan), according to the manufacturer’s instructions.

### Analysis of Gene Expression by Quantitative real-time PCR (qRT-PCR)

Quantitative real-time PCR was performed on an Applied Biosystems 7500 real-time PCR system using the SYBR Premix ExTaqTM Kit (TaKaRa Code: DRR041A, Japan). Sequence data, primers, and the PCR procedure used in this study were provided in our previous study [9]. All sequences of qRT-PCR amplification products were verified by sequencing. 18S rRNA was used as an internal control for expression analysis. Data were processed using the 2^−ΔΔCT^ method [28], [29].

The transcript levels of each BnNF-Y gene in each tissue were first normalized to those of the housekeeping gene 18S and then compared to the transcript levels of each time-point control to obtain the relative gene expression level (i.e., the levels of expression in the same untreated tissue at the corresponding time point). Each data point represents the mean ±SE of three independent experiments. For the figures S1-S5, the transcript levels of each *BnNF-Y* genes were first normalized to those of the housekeeping gene 18S and then compared to the levels in the 0-h leaf control.

### Statistical Analysis and Correlation Analysis

Significant differences between three treated samples and three untreated controls (same tissue and time-point only) are indicated by a single (P<0.05) or double (P<0.01) asterisk, according to Dunnett’s method of one-way ANOVA in SPSS 18.0 (SPSS Corp., Chicago, IL, USA). Figures were created using Sigma Plot 10.0 (Systat Software, Inc. Germany). Correlation analysis of expression patterns was determined using relative gene expression levels from Fig. S1, Fig. S2, and Fig. S3. The regression coefficient was calculated in Microsoft Excel.

### Measurements of Relative Water Content (RWC) from Drought-Stressed Plants

RWC measurements under PEG treatments were conducted as previously described [20].

### Isolation of *NF-Y* promoters and Identification of their regulatory elements

To identify the promoter regions of *NF-Ys* from canola, each *BnNF-Y* coding region was used as a query in a BLASTN search against the *Brassica* database (http://brassicadb.org/brad/index.php), which includes *B. rapa* and *B. oleracea* sequences, and the highest hits were identified. Based on *BnNF-Y* coding sequences and the promoter sequences of the retrieved orthologous *Brassica NF-Y*s, most promoter regions of *BnNF-Y* sequences could be amplified with the primers listed in Table S1. Others promoter regions were amplified using a Genome Walking Kit (TaKaRa Code: D316, Table S1). In addition, the promoters of *Arabidopsis NF-Y* sequences were downloaded from the Plant Promoter Database (http://133.66.216.33/ppdb/cgi-bin/index.cgi). Sequences ∼1000****bp upstream of the ATG start codon of each gene were retrieved from the genome, and known elements were identified using Plant *Cis*-element Regulatory DNA Elements (PLACE) (http://www.dna.affrc.go.jp/PLACE/) and PlantCARE (http://bioinformatics.psb.ugent.be/webtools/plantcare/html/).

### Phylogenetic tree analysis

The phylogenetic tree based on the promoter regions of plant NF-Ys was constructed using the neighbor-joining method implemented in MEGA software (version 4.1) with the following parameters: Jones–Taylor–Thornton (JTT) model and 1,000 bootstrap replicates.

## Results

### Expression Patterns of *BnNF-Y* Genes in the Leaves and Roots of Canola in Response to Salinity Stress

**Figure 1:**
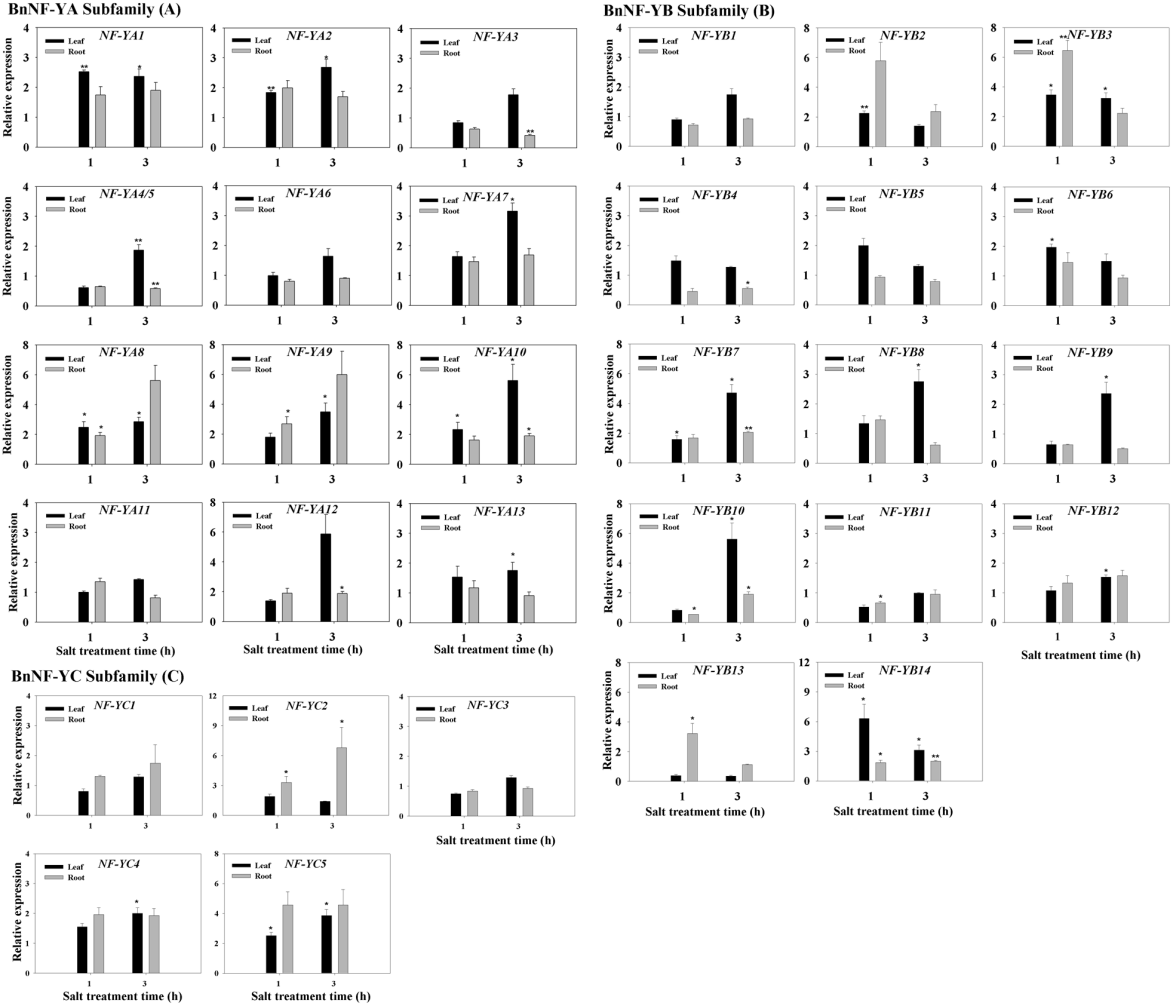
Expression pattern of the *BnNF-Y* genes in the leaves and roots of plants subjected to salinity stress. The expression of *BnNF-YA* (A), *BnNF-YB* (B), and *BnNF-YC* (C) genes in the leaves and roots of plants exposed to 150****mM NaCl. Three-week-old canola seedlings were treated with 150****mM NaCl for 1 and 3****h. Total RNA was extracted from leaves (L) and roots (R) for quantitative PCR (qRT-PCR) analysis. Transcript levels of each BnNF-Y were first normalized to those of the housekeeping gene 18S and then compared to each time-point control (i.e., the level of untreated sample at the corresponding time point). Each data point represents the mean ±SE of three independent experiments. Significant differences between treated samples and untreated controls (same tissue only) are indicated by a single (P<0.05) or double (P<0.01) asterisk, according to Dunnett’s method of one-way ANOVA in SPSS. Expression levels of untreated samples (C, 1-h or 3-h leaf and root samples) were arbitrarily set to 1.0.

We previously identified BnNF-Y TF families in canola (*B. napus* L.) [9]. Since numerous reports have demonstrated that plant NF-Ys function in abiotic stress responses [13], [15], we examined whether BnNF-Ys had similar roles. To investigate whether *BnNF-Y* genes were responsive to salt stress, we monitored the transcript levels of all 33 *BnNF-Y*s in both the leaves and roots up to 3****h of exposure to high salinity. First, we quantified the transcript levels of each *BnNF-Y* gene in different tissues and expressed these levels relative to those in 0-h untreated leaves. In this study, a gene was considered to be induced or down-regulated if its expression levels were both significantly different from those of an untreated control and >2-fold higher or lower than the control values, respectively. As shown in Fig. S1A, S2A and S3A, in the absence of any stress, the expression levels of most *BnNF-YA* subfamily members were not significantly altered during a 3-h observation period, except for *BnNF-YA4*/*5*, *BnNF-YA11*, *BnNF-YA12,* and *BnNF-YA13.* The transcription levels of *BnNF-YA4*/*5* (*BnNF-YA4* and *BnNF-YA5* are alternative splice variants, with *BnNF-YA4* being 11 amino acids shorter than *BnNF-YA5*) [9], and *BnNF-YA11* were up-regulated in the 3-h root samples, while levels of *BnNF-YA12* and *BnNF-YA13* were down-regulated in 1-h leaf samples. This implies that these *BnNF-YA*s are subject to circadian clock regulation. To minimize the effect of circadian-mediated changes, we recalculated the expression levels by dividing the expression level of each gene to the corresponding gene expression level in the untreated control at each time point and comparing values in the same tissues. As shown in Fig. 1A, the transcript levels of *BnNF-YA1*, *BnNF-YA2, BnNF-YA8* and *BnNF-YA10* in leaves were higher than those of the control after 1****h of NaCl treatment and remained elevated up to 3****h of treatment, whereas those of, *BnNF-YA7* and *BnNF-YA9* in leaves were higher than those of the control up to 3****h of treatment. In roots, two *BnNF-YA* subfamily members (*BnNF-YA9* and *BnNF-YA10*) were induced at 1****h or 3****h. Most salt-responsive *BnNF-YA*s were only moderately up-regulated (2∼4 fold) upon exposure to salt stress, whereas *BnNF-YA3* and *BnNF-YA4/5* were slightly down-regulated in roots upon NaCl treatment.

We found that the expression of only two members of the *BnNF-YB* subfamily (i.e., *BnNF-YB10*, and *BnNF-YB13*) increased during a 3-h period in the absence of any treatments (Fig. S1B, S2B, and S3B). In leaves, the transcript levels of six *BnNF-YB*s (i.e., *BnNF-YB3, BnNF-YB7*, *BnNF-YB8*, *BnNF-YB9, BnNF-YB10,* and *BnNF-YB14*) were elevated after 1****h or 3****h of NaCl stress (Fig. 1B). In roots, NaCl treatments increased the transcript levels of *BnNF-YB3*, *BnNF-YB13*, and *BnNF-YB14.* Our results show that the levels of *BnNF-YB1*, *BnNF-YB5*, *BnNF-YB6*, *BnNF-YB9*, *BnNF-YB11,* and *BnNF-YB12* transcripts were mostly not affected by salinity. Only the expression level of *BnNF-YB4* in the roots was decreased at 3****h.

None of the five *BnNF-YC* subunits was regulated by the biological clock, since the expression levels of these genes remained constant in the absence of any treatments (Fig. S1C, S2C, and S3C). Only *BnNF-YC5* was induced in the leaf samples both at the 1- and 3-h time-points under salinity (Fig. 1C). *BnNF-YC2* transcripts were more abundant in roots subjected to NaCl treatments than in untreated roots.

### Expression Patterns of *BnNF-Y* Genes in Leaves and Roots of Canola in Response to drought Stress

**Figure 2:**
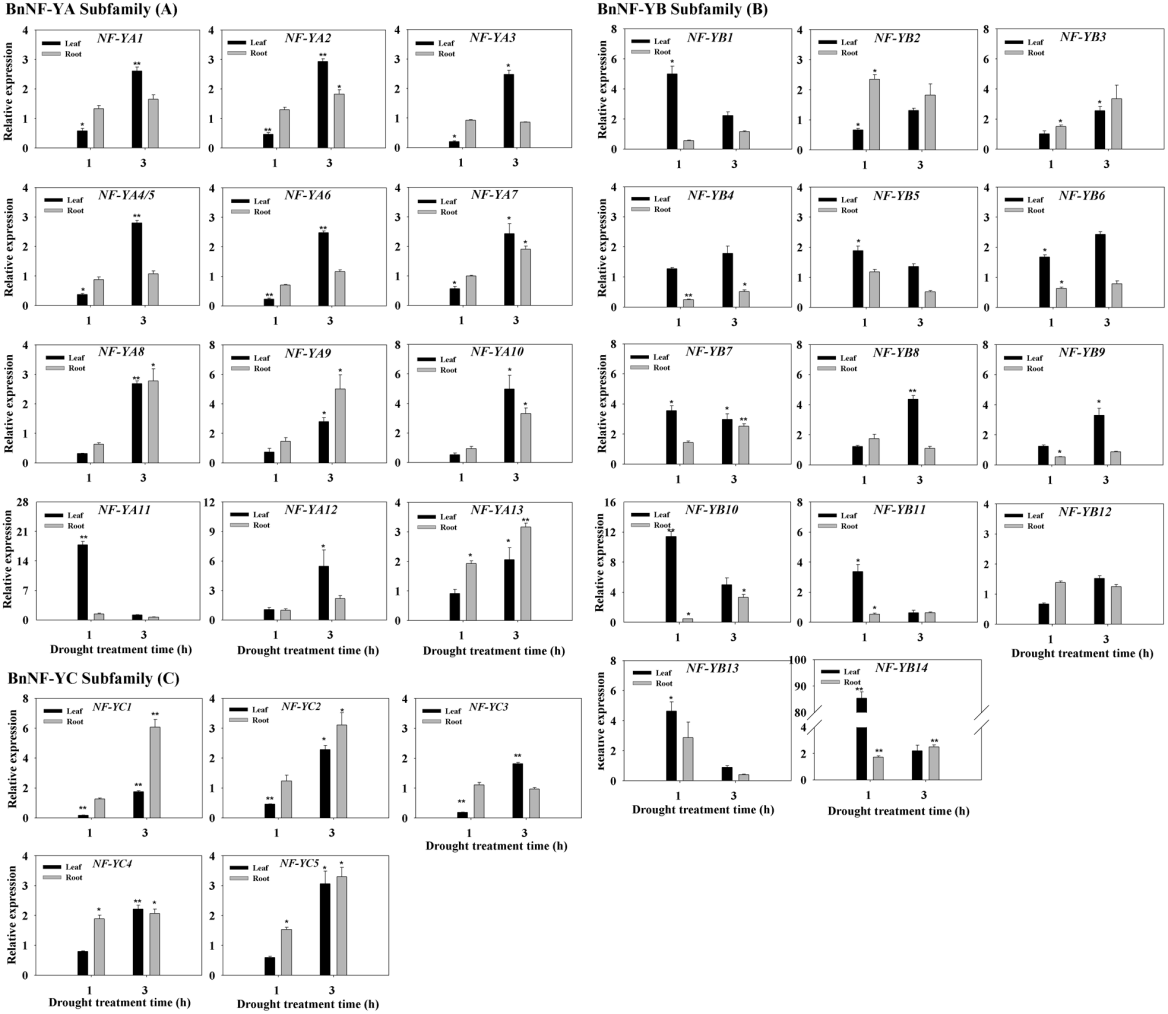
Expression pattern of the *BnNF-Y* genes in plants subjected to drought stress. The expression of *BnNF-YA* (A), *BnNF-YB* (B), and *BnNF-YC* (C) genes in the leaves and roots of plants exposed to 15% (w/v) PEG6000. Three-week-old canola seedlings were treated with 15% (w/v) PEG-6000 for 1 and 3****h. Total RNA was extracted from leaves (L) and roots (R) for quantitative PCR (qRT-PCR) analysis. Transcript levels of each BnNF-Y were first normalized to those of the housekeeping gene 18S and then compared to each time-point control. Each data point represents the mean ±SE of three independent experiments. Significant differences between treated samples and untreated controls (same tissue only) are indicated by single (P<0.05) or double (P<0.01) asterisks, according to Dunnett’s method of one-way ANOVA in SPSS. Expression levels in untreated samples (CK, 1-h or 3-h leaf and root samples) were arbitrarily set to 1.0.

As shown in Fig. 2, all *BnNF-YA* members were responsive to PEG6000 treatments, which mimic drought stress, in leaf tissues. A 15% PEG treatment generally caused a 72% relative water content loss within 24****h (Table S2). The expression levels of *BnNF-YA10*, *BnNF-YA11* and *BnNF-Y12* in leaves were dramatically up-regulated to more than four-fold the levels observed in the untreated control (Fig. 2A). *BnNF-YA8*, *BnNF-YA9*, *BnNF-YA10* and *BnNF-YA13* were induced in roots under drought stress. Four *BnNF-YA*s (*BnNF-YA8*, *-YA9*, *-YA10,* and *-YA13*) responded to drought stress both in leaves and roots, particularly up to 3****h of treatment. The expression levels of five *BnNF-YA*s (*BnNF-YA1*, *BnNF-YA2*, *-YA3*, *-YA4/5,* and -*YA*6) decreased in leaves at 1****h and increased at 3****h.

Most members of the *BnNF-YB* subfamily also responded to PEG treatments, except for *BnNF-YB12*, while the expression level of *BnNF-YB14* increased 80-fold in leaves after 1****h of treatment (Fig. 2B). As in the *BnNF-YA* subfamily, the transcripts of *BnNF-YB* were induced more strongly in leaves than in roots, since only 3 of 14 *BnNF-YB* members (*BnNF-YB7, BnNF-YB10,* and *BnNF-YB14*) were induced in roots subjected to drought stress. Four BnNF-YBs (*BnNF-YB4*, *-B9*, *-B10* and *-B11*) exhibited decreased expression in the roots after 1****h of treatment.

The transcript levels of four *BnNF-YC* members (*BnNF-YC1, BnNF-YC2, BnNF-YC4,* and *BnNF-YC5)* were elevated in both the leaf and root samples up to 3****h of drought treatment, while those of three *BnNF-YC*s (*BnNF-YC1*, *BnNF-YC2,* and *-YC3*) were initially inhibited in leaves at the 1-h time point (Fig. 2C).

### Expression Patterns of *BnNF-Y* Genes of Canola in Leaves and Roots in Response to ABA Stress

**Figure 3:**
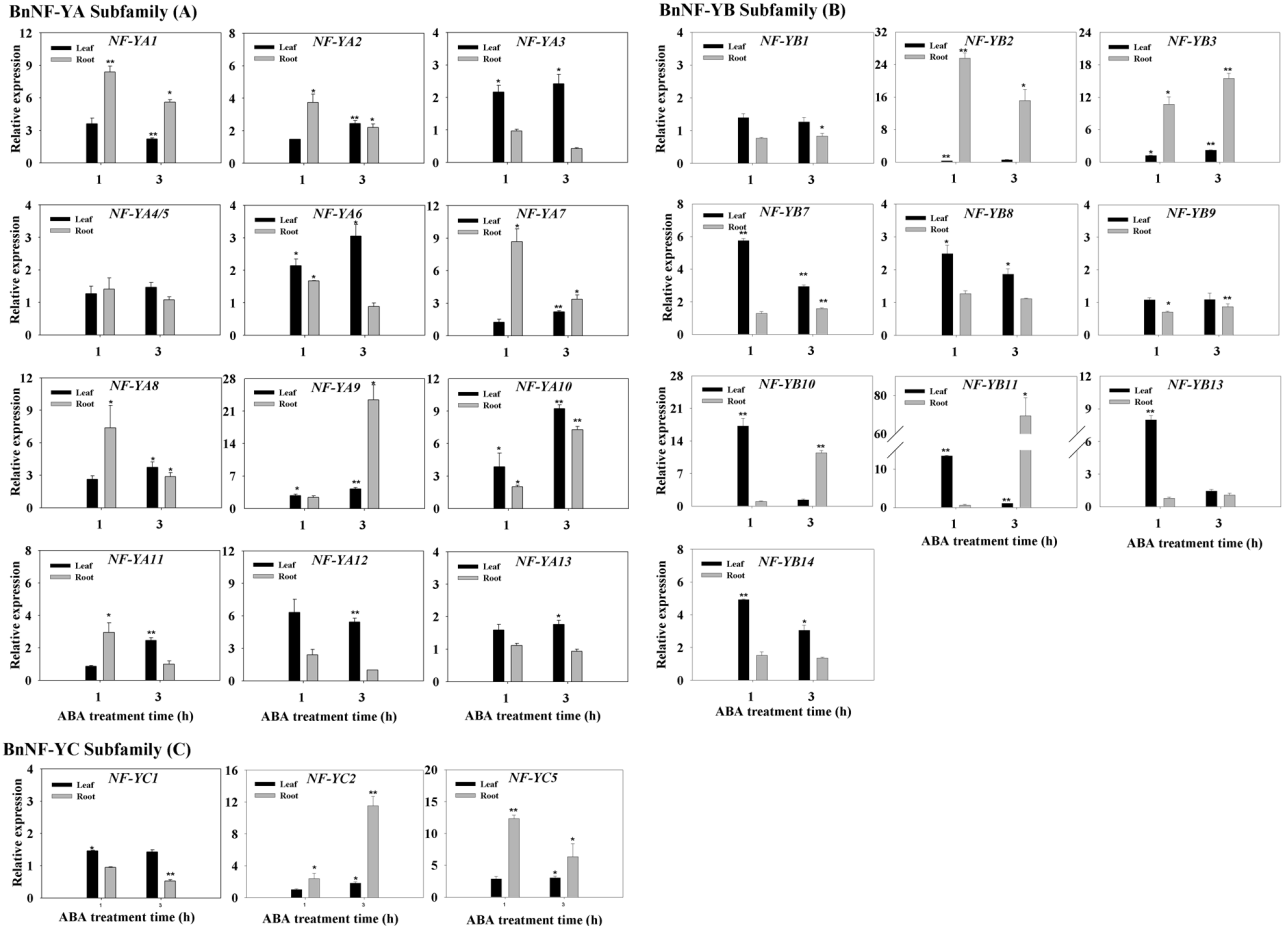
Expression pattern of the *BnNF-Y* genes in the leaves and roots of plants exposed to 100 µM ABA. Transcript levels of *BnNF-Y (*A), *BnNF-YB* (B), and *BnNF-YC* (C) genes after exposure to 100****µM ABA. Three-week-old canola plantlets were treated with 100****µM ABA for 1****h and 3****h. Total RNA was extracted from leaves and roots for quantitative PCR (qRT-PCR) analysis. Transcript levels of each BnNF-Y were first normalized to those of the housekeeping gene 18S and then compared to levels at each time point in the control. Each data point represents the mean ±SE of three independent experiments. Significant differences between treated samples and untreated controls (same tissue only) are indicated by a single (P<0.05) or double (P<0.01) asterisk, according to Dunnett’s method of one-way ANOVA in SPSS. Expression levels in untreated samples (CK, 1-h or 3-h leaf and root samples) were arbitrarily set to 1.0.

Plants respond to abiotic stresses via both the ABA-dependent and ABA-independent pathways. Some NF-Ys were reported to be involved in ABA-dependent pathways, whereas others were not [11], [13], [15]. To determine whether canola NF-Ys function via the ABA pathway, we further examined the expression profiles of *BnNF-Y*s that were found to be responsive to salinity or drought stress (i.e., all *BnNF-YA* members and most *BnNF-YB*s and *BnNF-YC*s; Figs 1 and 2) under ABA treatments. All *BnNF-YA*s, except for *BnNF-YA4/5* and *BnNF-YA13,* were responsive to 3-h of ABA treatment (Fig. 3A). The transcript levels of *BnNF-YA1*, *-YA2*, *-YA3*, *-YA6*, *-YA7*, *-YA8*, *-YA9*, *-YA10* and *BnNF-YA11* were elevated at 1****h. Also, most NaCl- or PEG- responsive *BnNF-YB* members were induced by ABA treatments, with the exception of *BnNF-YB1* and *BnNF-YB9* (Fig. 3B). The transcript level of *BnNF-YB2* was inhibited in leaves after 1****h of ABA treatment. Interestingly, several *BnNF-YB*s (*BnNF-YB2, -YB3*, *-YB7, -YB10*, *-YB11, -YB13,* and *-YB14*) were greatly up-regulated by ABA treatment. Amongst the *BnNF-YC* members, only the transcripts of *BnNF-YC2* and *BnNF-YC5* were increased in response to ABA treatment, and the upregulation was greater in the roots than in the leaves (Fig. 3C). The transcript levels of *BnNF-YC1* remained almost constant from 1****h to 3****h of ABA treatment in all tissues examined.

To compare the gene expression levels of different *BnNF-Y*s in canola, the expression level of each *NF-Y* gene in the 0-h leaf or root samples (arbitrarily chosen as baseline in the real-time PCR analysis) was also determined by semi-quantitative RT-PCR analysis (35 cycles; Fig. S4). The *BnNF-Y*s showed diverse expression profiles. Interestingly, most *BnNF-Y*s were expressed at similar levels in 0-h leaves and 0-h roots.

### Correlation between the gene expression patterns of *BnNF-Y* genes

**Figure 4:**
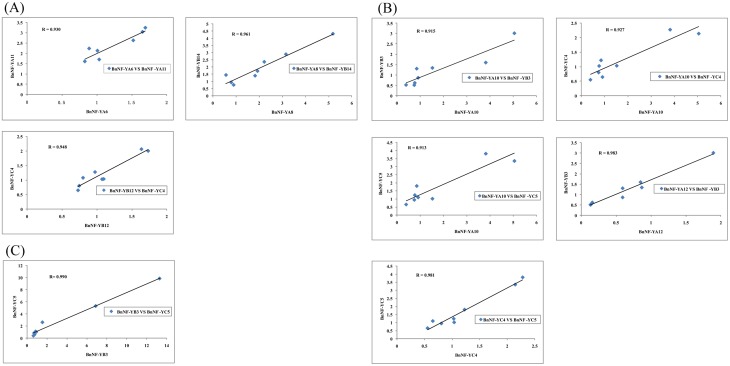
Correlation in expression levels of the three BnNF-Y subfamily members. Correlated gene expression under salinity (A); drought (B); and ABA (C) treatments. Relative expression levels including 8 data points (relative NF-Y gene expression levels with and without treatments at 1****h or 3****h compared to the same 0-h samples) are used for each gene according to Fig. S1, S2, and S3. These data were fitted using linear regression analysis.

Since NF-Y TFs are known to form functional complexes in plants [30], [31], one would expect that NF-Ys with correlated expression profiles would be involved in the same regulatory processes under abiotic stress conditions. Extensive correlations were found to exist in the expression profiles of *BnNF-Ys* not only within the same subfamily, but also between the three subfamilies. The expression profiles of *BnNF-YA6* and *BnNF-YA11,* of *BnNF-YA8* and *BnNF-YB14,* and of *BnNF-YC4* and *BnNF-YB12* were highly correlated under NaCl treatment (R = 0.93, 0.948 and 0.961, respectively. Fig. 4A). Under drought stress, the expression of *BnNF-YA10* and *-YB3, -YC4,* or *-YC5,* of *-YA12* and *-YB3,* and of *-YC4* and *-YC5* were highly correlated (R = 0.915, 0.927, 0.913, 0.983, and 0981, respectively. Fig. 4B). Under ABA treatments, *BnNF-YB3* and *BnNF-YC5* showed very similar expression profiles (R = 0.99. Fig. 4C).

### The Promoters of *NF-Y* Genes Contain Diverse Stress-responsive Elements

**Figure 5:**
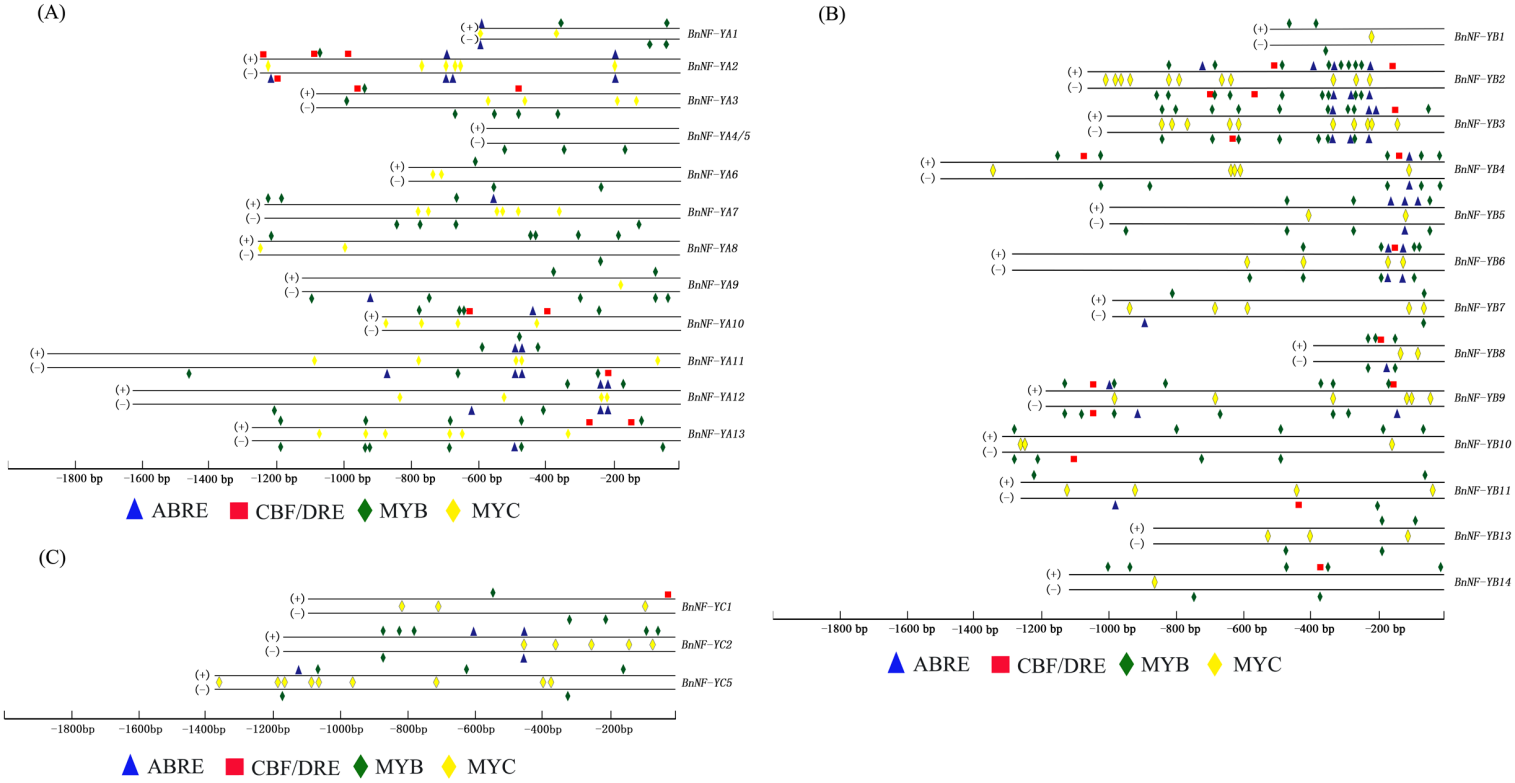
Diagrammatic representation of stress-related elements in the *BnNF-*Y promoters. Elements in the *BnNF-*YA (A), *BnNF-YB* (B), and *BnNF-YC* (C) promoters. Symbols above the top line indicate elements that are in the forward orientation, and those below the bottom line indicate elements in the reverse orientation. MYC(CANNTG) sequences are between the double lines, to indicate their palindromic nature. Numbers are the distance in nucleotides between the sequences and ATG start codon.

Since most BnNF-Y members were found to be regulated by abiotic stress, we sought to identify the promoter sequences of their corresponding genes and to identify stress-relative *cis*-elements. We cloned the promoters of 12 *BnNF-YAs*, 13 *BnNF-YBs*, and 3 *BnNF-YCs* that were responsive to one or more of the three treatments used above (i.e., NaCl, PEG6000, and ABA). Using a BLASTN search and genome walking, we successfully acquired all the promoter sequences, most of which consisted of around 1000****bp upstream of the ATG start codon. However, we were unable to acquire the promoter sequences of two *BnNF-YA* members (*BnNF-YA4/5* and *BnNF-YA6*) and two *BnNF-YB* members (*BnNF-YB1* and *BnNF-YB8*) longer than 1000****bp. We then identified *cis*-elements in these promoters using PLACE and PlantCARE software (Fig. 5), focusing on stress-related *cis*-elements, such as ABRE, CBF/DRE, MYB, and MYC. The promoters of each of the above-mentioned *BnNF-Y*s included at least two types of *cis*-elements, with the exception of the truncated *BnNF-YA4*/*5* promoter sequences. All of the promoters, except for the promoter of *BnNF-YA4*/*5,* harbored MYB and MYC elements. More than half of the *BnNF-YA* and *BnNF-YC* promoters harbored at least one ABRE element, while only a few BnNF-Ys had DRE elements. We also constructed *Arabidopsis cis*-elements in the homologous *NF-Y* family and compared the arrangement of *cis*-elements in canola and *Arabidopsis*. Most canola *NF-Y* members had analogous *cis*-elements in *Arabidopsis*, but the number and arrangement of *cis*-elements differed (Fig. S5).

### Canola promoter sequences showed greater similarities with those of *B. rapa* than with those of *Arabidopsis thaliana*

**Figure 6:**
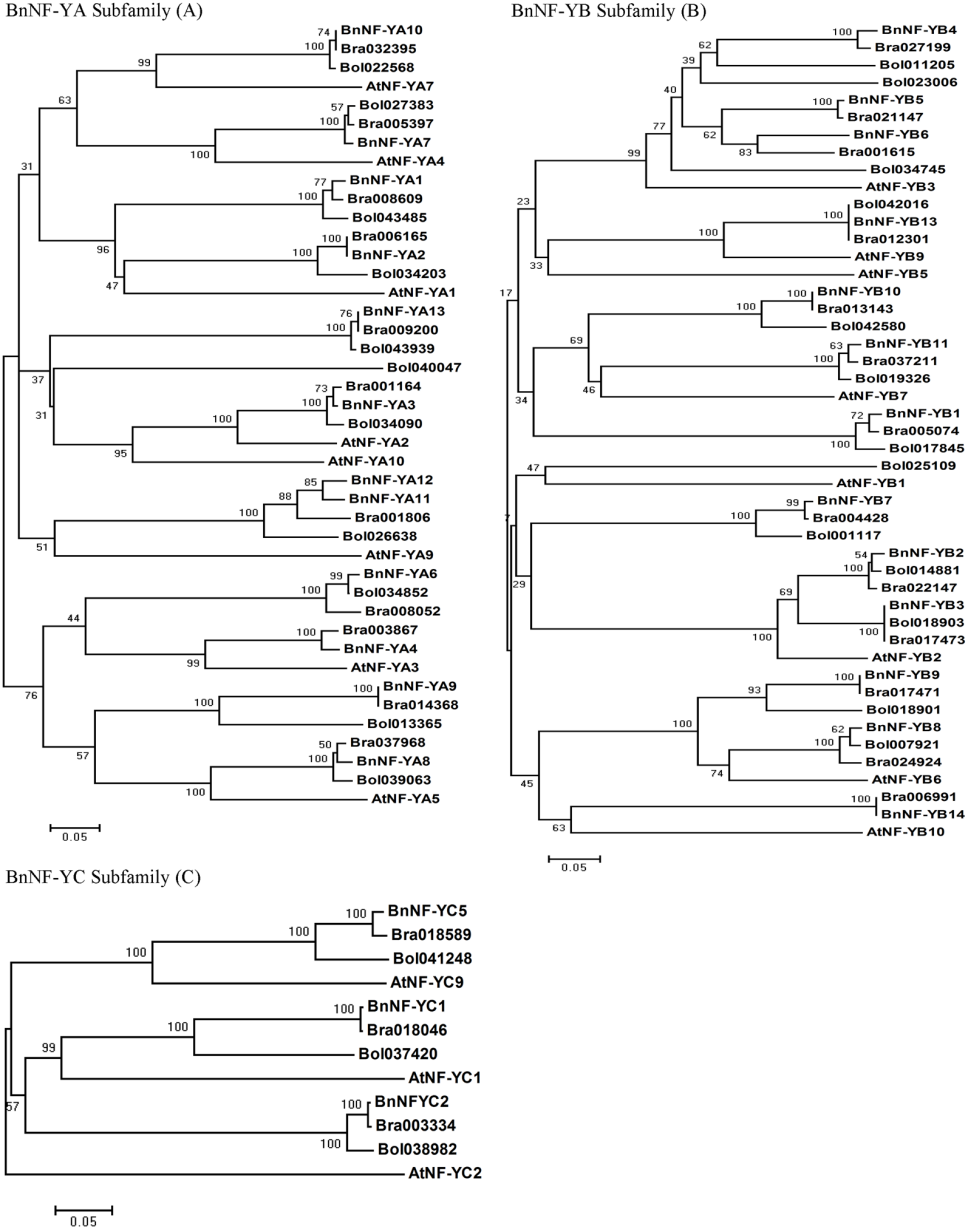
Phylogenetic trees of *Brassica* and *Arabidopsis* NF-Y families based on the nucleotide sequences in the promoter regions. The phylogenetic trees were constructed by the neighbor-joining method implemented by MEGA software, version 4.1. The numbers at each branch point represent the bootstrap scores (1,000 replicates). A branch with a bootstrap score of below 50 was usually considered unreliable. a Phylogenetic tree of *Brassica* and *Arabidopsis* NF-YA promoters. Due to low levels of nucleotide similarity, *BnNF-YA4* homologues in *B. oleracea* were not included here. b Phylogenetic tree of *Brassica* and *Arabidopsis* NF-YB promoters. Due to low levels of nucleotide similarity, *BnNF-YB14* homologues in *B. oleracea* were not included here. c Phylogenetic tree of *Brassica* and *Arabidopsis* NF-YC promoters. Bol and Bra represent *B. oleracea* and *B. rapa*, respectively. The numbers after the species abbreviation correspond to the individual gene name. Most promoters were at least 1000****bp in length, except for those of *BnNF-YA1* (595****bp), *BnNF-YA4/5* (577), *BnNF-YA6* (806), *BnNF-YA10* (892), *BnNF-YB1* (519), *BnNF-YB7* (988), *BnNF-YB8* (391), and *BnNF-YB13* (867) and their homologues in *B. oleracea*, *B. rapa,* and *Arabidopsis*.

We previously showed that canola NF-Ys were more similar to those of the dicot *Arabidopsis* than to those of the monocot *Oryza sativa* (rice) based on putative amino acid sequences. In this study, we also used the promoter sequences to further explore the phylogenetic relationship amongst members of this family. All canola promoters grouped with promoters of their own homologs and formed clades with the corresponding subfamily members in the other species (Fig. 6). The promoter sequence of each canola *NF-Y* usually had the highest level of identity with *B. rapa,* and the lowest with *Arabidopsis,* with the exceptions of *BnNF-YA6*, *BnNF-YB2* and *BnNF-YB8*. The promoter regions of these three *BnNF-Ys* were more similar to those of *B. oleracea* than to those of *B. rapa*.

## Discussion

The fact that multiple members of the NF-Y family exist in plant genomes implies that these proteins have redundant or differentiated functions. In this study, we extensively explored the response of *BnNF-Y*s to abiotic stresses through qRT-PCR analysis. The expression profiles of the *BnNF-Y* TF family provide insight into their roles during the abiotic stress response.

It is not surprising that most identified *BnNF-YA*s are involved in salinity, drought, or ABA treatments, as most previously reported stress-responsive plant NF-Y members belong to the NF-YA subfamily [15]. Quantitative RT-PCR analysis indicated that transcripts of *Arabidopsis NF-YA1* were increased by salinity, PEG, and ABA treatments. It was previously found that overexpression of *Arabidopsis NF-YA1* rendered the transgenic plants more sensitive than the control to a 200****mM NaCl treatment, whereas the corresponding *Arabidopsis* RNAi plants grew better than the control under these conditions [15]. *BnNF-YA1* and *BnNF-YA2*, which showed the highest level of similarity with *Arabidopsis NF-YA1* [9], were induced by salinity, PEG, and ABA treatments. Overexpression of *Arabidopsis NF-YA2*, *NF-YA7,* and *NF-YA10* caused a dwarf phenotype and enhanced the plant’s resistance to multiple abiotic stresses [25]. We previously showed that BnNF-YA3, BnNF-YA13, and BnNF-YA14 formed a clade with AtNF-YA2 and AtNF-YA10 [9]. Expression of *Arabidopsis NF-YA5* was up-regulated up to 14 days of drought treatment or within 24****h of ABA treatment [13]. The ectopic expression of *NF-YA5* in *Arabidopsis* increased the plant’s drought tolerance [13]. BnNF-YA8 or BnNF-YA9, which clustered with *Arabidopsis* NF-YA5, exhibited similar expression patterns up to 3****h of PEG treatment. Using an inducible estradiol system, the overexpression of *NF-YA1*, *5*, *6*, or *9* was shown to result in increased ABA sensitivity during seed germination [32]. One NF-YA homolog, *GmNF-YA3,* from soybean was also shown to be involved in the abiotic stress response. Transcripts of *GmNF-YA3* were induced after as little as 0.5****h of NaCl, ABA, or cold treatment, and GmNF-YA3 showed a high level of similarity with *Arabidopsis* NF-YA3, 5, 6, and 8. Of the reported members of the plant NF-YA subfamily, the transcript levels of several members (including *NF-YA1, 5, 6,* and *9*) were affected by ABA, implying that these genes regulate the plant’s response to abiotic stress via an ABA-dependent pathway. This may also be the case for *BnNF-YA1*, *BnNF-YA8,* and *BnNF-YA9,* which exhibit similar expression patterns under salinity, drought, or ABA stress.

AtNF-YB1 and its maize homologue ZmNF-YB2 were shown to enhance drought resistance, and maize plants overexpressing *ZmNF-YB2* performed well in the field [11]. Barley *HvNF-YB5,* similar to *Arabidopsis* NF-YB1, showed around a 70-fold expression increase under salinity treatment [20]. *Triticum aestivum* (wheat) *TaNF-YB2*, homologous to *Arabidopsis* NF-YB1, was also upregulated in response to drought stress in the leaves [18]. *BnNF-YB1*, which is homologous to *Arabidopsis NF-YB1*, was induced only by drought in our study. *Arabidopsis NF-YB2* was first found to be up-regulated by NaCl, mannitol, or cold (4°C) treatment [17]. Interestingly, *NF-YB2* was identified as the top third gene to respond to osmotic stress. The expression of poplar *NF-YB7* increased during a 15-d PEG or ABA treatment [33]. Transgenic *Arabidopsis* plants overexpressing poplar *NF-YB7* exhibited an increased germination rate, root length, and photosynthetic rate under drought stress. Poplar NF-YB7 showed a high level of amino acid sequence identity with *Arabidopsis* NF-YB3, while *Arabidopsis nf-yb3* mutant plants were more sensitive than the wild type to environmental stimuli. Canola BnNF-YB2 and BnNF-YB3, similar to *Arabidopsis* NF-YB2, were found to be responsive to NaCl and ABA treatments in this study. The expression of several poplar *NF-YBs*, such as *NF-YB6*, *NF-YB11*, and *NF-YB13,* was altered in the leaves of plants treated with PEG-6000 [19], while the expression of *BnNF-YB7* and *BnNF-YB14* (high identity with Poplar NF-YB11 and NF-YB13 in terms of the conserved core domain) was also up-regulated in the leaves under PEG or NaCl treatment (Fig. 1 and Fig. 2).

Previous studies revealed that a few NF-YC members were involved in the stress response [21], [22]. One NF-YC member, *NF-YC2,* from both *Arabidopsis* and *Nicotiana tabacum* (tobacco), was found to be activated by photooxidative stress [21]. This gene responded to multiple stresses, including heat, cold, and drought. In the same study, several *Arabidopsis NF-YC* members were shown to be induced by abiotic stress. The transcript levels of *Arabidopsis NF-YC3* and *NF-YC4* were increased under drought stress, while *NF-YC4* was also induced by cold. Furthermore, *Arabidopsis* NF-YC2 formed a complex with NF-YB3, bZIP28, and NF-YA4 and was shown to play a role during endoplasmic reticulum stress [30]. *PwHAP5* (*Picea wilsonii*) was up-regulated by NaCl, dehydration, and ABA treatment [22]. Ectopic expression of *PwHAP5*, which is homologous to *Arabidopsis* NF-YC2, improved salinity tolerance in plants. In our previous study, we showed that BnNF-YC2 and BnNF-YC5 grouped with NF-YC2 and NF-YC3 [9] and, like these homologs in *Arabidopsis*, responded to salinity and drought stress.

Since plants have so many NF-Ys, functional redundancy seems inevitable. *Arabidopsis* NF-YB2 and NF-YB3 function additively in the long-day flowering pathway [34]. LEAFY COTYLEDON 1 (LEC1) and LEAFY COTYLEDON1-LIKE (L1L) were shown to be involved in embryo development [35], [36]. Many of the BnNF-Ys characterized in our study were responsive to salinity, drought, or ABA treatment. Some of these members in the same subfamily were clustered in the same phylogenetic clade based on their promoter sequences, such as BnNF-YA1 and BnNF-YA2 (Fig. 6). Further evidence is needed to confirm the roles of these proteins during the abiotic stress response. It would also be interesting to explore whether these proteins from the three subfamilies combine to form trimeric complexes, as reported in yeast and animal systems [37]. It is not known whether the NF-Y complex always has fixed components or whether the components differ under different conditions. Even though extensive correlations in the expression patterns of *TaNF-Y* subfamily members were identified [18], no three members from the three different BnNF-Y subfamilies exhibited the same expression pattern under all three treatments in our study. This phenomenon implies that the canola BnNF-Y complex does not always consist of the same monomers under different conditions.

The plant’s response to abiotic stress involves the transcriptional regulation of genes via their *cis*-regulatory elements. ABRE, MYB, and MYC elements are known to be involved in the ABA-dependent stress pathway, while the DRE element plays a role in the ABA-independent pathway [38]–[40]. Two ABRE elements were identified in the promoter region of *Arabidopsis* drought-responsive *NF-YA5,* which functioned in an ABA-dependent manner [13]. Interestingly, *GmNF-YA3*, a homolog of *NF-YA5*, harbored an additional DRE *cis*-acting element in its promoter, which suggests that soybean NF-YA3 may be involved in both pathways [16]. A previous study of the global expression patterns of rice plants subjected to various abiotic stresses identified more ABRE and DRE elements in the promoter regions of genes responsive to both drought and salinity than in those specifically responsive to drought or salinity stress [41]. In our study, the promoter regions of *BnNF-YA11* and *BnNF-YA12*, which were strongly induced by salinity and drought stress, each harbored 4 ABRE elements. Canola *NF-YB2* and *NF-YB3* each possessed at least 5 ABRE elements and were strongly up-regulated by ABA treatments. *BnNF-YC2,* which contains 2 ABRE elements in its promoter region, was strongly induced by ABA stress. In contrast, the promoter regions of canola *NF-YB11* and *NF-YB14,* which were also strongly induced by ABA or drought treatment, had few DRE or ABRE elements, but several MYB or MYC elements, suggesting that MYB or MYC play roles in the abiotic stress response.

According to the well-known triangle theory [42], [43], canola, an allopolyploid, originated from the hybridization of *B. rapa* (the A genome) and *B. oleaacea* (the C genome), while all *Brassica* species basically arose from common *Arabidopsis* ancestors. Based on our NF-Y stress-related *cis*-element analysis and promoter sequence alignments, the upstream regulatory regions of *NF-Y* sequences of canola were found to be similar to those of *Arabidopsis* (Fig. 5 and 6). Through extensive comparisons based on nucleotide sequences, homoeologous segments conserved in canola and *Arabidopsis* were found to exhibit perfect collinearity [44]. Our study revealed that the promoters of canola *NF-Ys* were more similar to those of *B. rapa* than to *B. oleracea*. Canola (*B. napus*) was proposed to have multiple origins, and natural canola species were found to be more closely related to *B. rapa* species than to *B. oleracea* species, according to an Restriction fragment length polymorphisms (RFLP) analysis of nuclear, chloroplastic, and mitochondrial DNA [45]. A recent study found that two canola self-incompatibility genes (S-locus glycoproteins, SLGs) and an S-locus receptor kinase (SRK) had higher levels of amino acid sequence identity with their *B. rapa* homologues than with those from *B. oleracea* [46], supporting the notion that a subset of canola sequences are more closely related to *B. rapa* than to *B. oleracea*.

In conclusion, this study represents an extensive evaluation of BnNF-Y family members under salinity, drought, or ABA stress. The results presented here offer a useful foundation for further studies of BnNF-Y proteins under abiotic stress conditions. Several *BnNF-Y* members in each subfamily showed similar expression patterns, indicating that these genes may have redundant functions. Members of different families were found to have similar expression patterns, suggesting that BnNF-Ys form a heterocomplex. Our BnNF-Y promoter analysis shows that multiple BnNF-Y members contain abiotic stress-responsive elements and provides clues as to the evolution of BnNF-Ys in *Brassica* species.

## Supporting Information

Figure S1 **Expression pattern of** ***BnNF-Y*** **genes in the leaves and roots of plants subjected to salinity stress for 1 or 3 h.** The expression of *BnNF-YA* (A), *BnNF-YB* (B), and *BnNF-YC* (C) in the leaves and roots of plants exposed to 150****mM NaCl for the indicated periods of time. The transcript levels of each *BnNF-Y* gene were first normalized to those of the housekeeping gene 18S and then compared to the control (0-h level in the leaf). The expression levels of untreated samples (C, 0-h leaf samples) were arbitrarily set to 1.0. L, leaves; R, roots. CK, no treatment; Salt, NaCl treatment. Significant differences between different samples and 0-h samples (same tissue only) are indicated by a single (P<0.05) or double (P<0.01) asterisk, according to Dunnett’s method of one-way ANOVA in SPSS.
(DOC)

Figure S2 **Expression pattern of** ***BnNF-Y*** **genes exposed to osmotic stress.** The expression of *BnNF-YA* (A), *BnNF-YB* (B), and *BnNF-YC* (C) genes in the leaves of plants exposed to treatment with 15% (w/v) PEG-6000 for the indicated periods. The transcript levels of each *BnNF-Y* gene were first normalized to those of the housekeeping gene 18S and then compared to the levels in the 0-h leaf control. Expression levels in untreated samples (C, 0-h leaf samples) were arbitrarily set to 1.0. L, leaves; R, roots. CK, no treatment; Drought, PEG6000 treatment. Significant differences between different samples and 0-h samples (same tissue only) are indicated by a single (P<0.05) or double (P<0.01) asterisk, according to Dunnett’s method of one-way ANOVA in SPSS.
(DOC)

Figure S3 **Expression pattern of the** ***BnNF-Y*** **genes after exposure to 100 µM ABA.** The expression of *BnNF-YA* (A), *BnNF-YB* (B), and *BnNF-YC* (C) genes in the leaves and roots of plants exposed to 100****µM ABA. Transcript levels of each *BnNF-Y* were first normalized to those of the housekeeping gene 18S and then compared to levels at 0****h in the control (untreated) leaves. Expression levels in untreated samples (CK, 0-h leave samples) were arbitrarily set to 1.0. L, leaves; R, roots. CK, no treatment; ABA, ABA treatment. Significant differences between different samples and 0-h samples (same tissue only) are indicated by a single (P<0.05) or double (P<0.01) asterisk, according to Dunnett’s method of one-way ANOVA in SPSS.
(DOC)

Figure S4 **Semi-quantitative RT-PCR analysis of BnNF-Y expression in control leaves and roots.** RT-PCR analysis was performed on untreated leaf (L) and root (R) tissue samples. The 18S housekeeping gene and *BnNF-Ys* were amplified for 28 cycles and 35 cycles, respectively.
(DOC)

Figure S5 **Diagrammatic representation of NF-Y promoter regions.** *Arabidopsis* NF-YA (A), NF-YB (B), and NF-YC (C) promoter regions. Symbols above the top line indicate elements in the forward orientation, and those below the bottom line are in the reverse orientation. MYC(CANNTG) sequences are between the double lines, to indicate their palindromic nature. Numbers are the distance in nucleotides between the sequences and ATG start codon.
(DOC)

Table S1 **Primers for BnNF-Y promoters**. F indicates the PCR forward primer and R indicates the PCR reverse primer. Particularly, SP1, SP2, SP3 are three specific primers for Genome Walking.
(DOC)

Table S2 **Relative water content (RWC) of drought-stressed plants.** Plants were treated with 10%, 15% and 20% (w/v) PEG6000 solution for 24 h.
(DOC)
