# Supplementary material for: Multiple NUCLEAR FACTOR Y Transcription Factors Respond to Abiotic Stress in Brassica napus L
Source: PLoS One. 2014 Oct 30;9(10):e111354. doi: 10.1371/journal.pone.0111354 (PMC4214726; doi:10.1371/journal.pone.0111354)
Supplement: Figure S1 — Expression pattern of BnNF-Y genes in the leaves and roots of plants subjected to salinity stress for 1 or 3 h. The expression of BnNF-YA (A), BnNF-YB (B), and BnNF-YC (C) in the leaves and roots of plants exposed to 150mM NaCl for the indicated periods of time. The transcript levels of each BnNF-Y gene were first normalized to those of the housekeeping gene 18S and then compared to the control (0-h level in the leaf). The expression levels of untreated samples (C, 0-h leaf samples) were arbitrarily set to 1.0. L, leaves; R, roots. CK, no treatment; Salt, NaCl treatment. Significant differences between different samples and 0-h samples (same tissue only) are indicated by a single (P<0.05) or double (P<0.01) asterisk, according to Dunnett’s method of one-way ANOVA in SPSS. (DOC) [file pone.0111354.s001.doc]

**BnNF-YA Subfamily (A)**


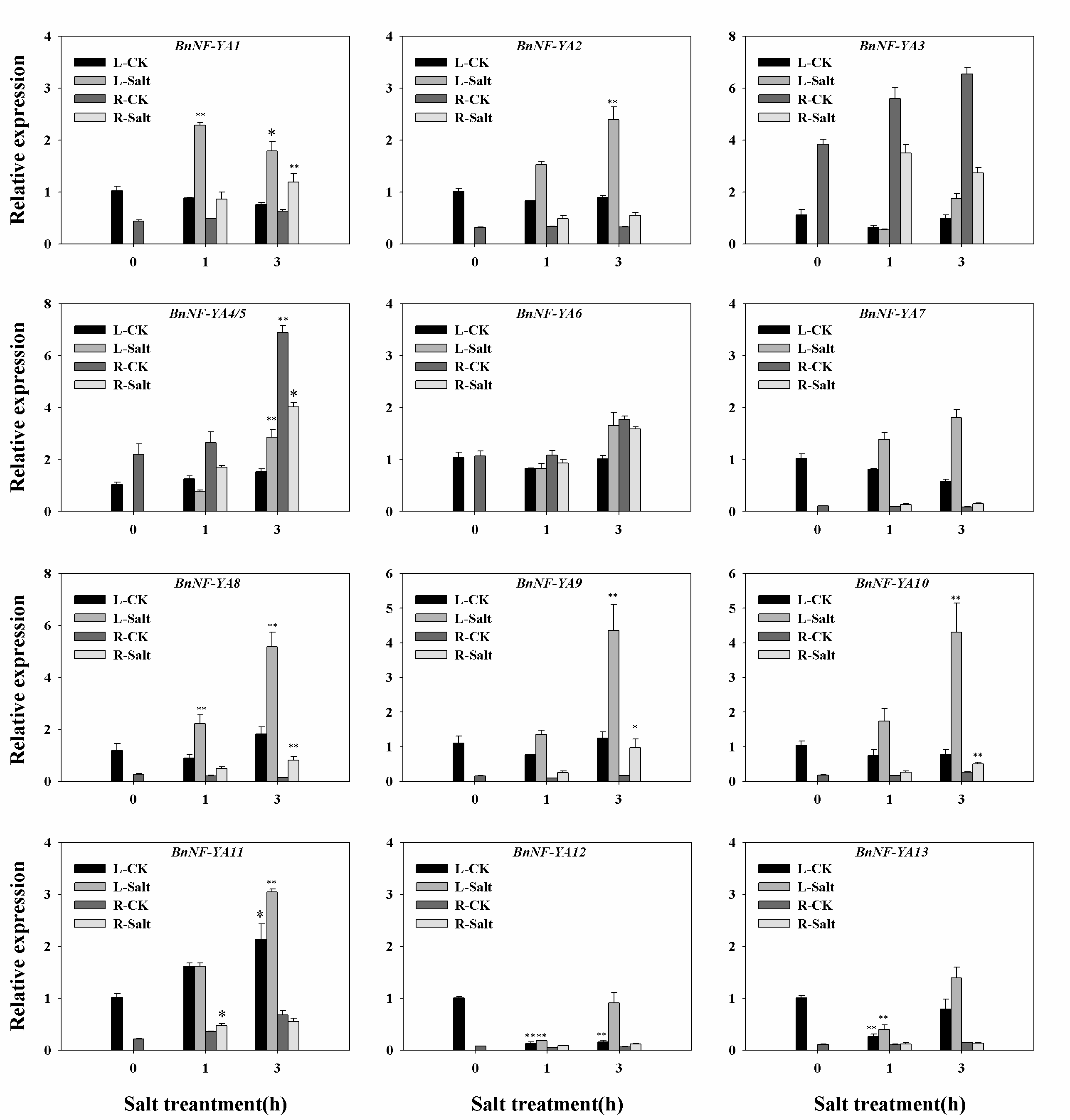


**BnNF-YB Subfamily (B)**


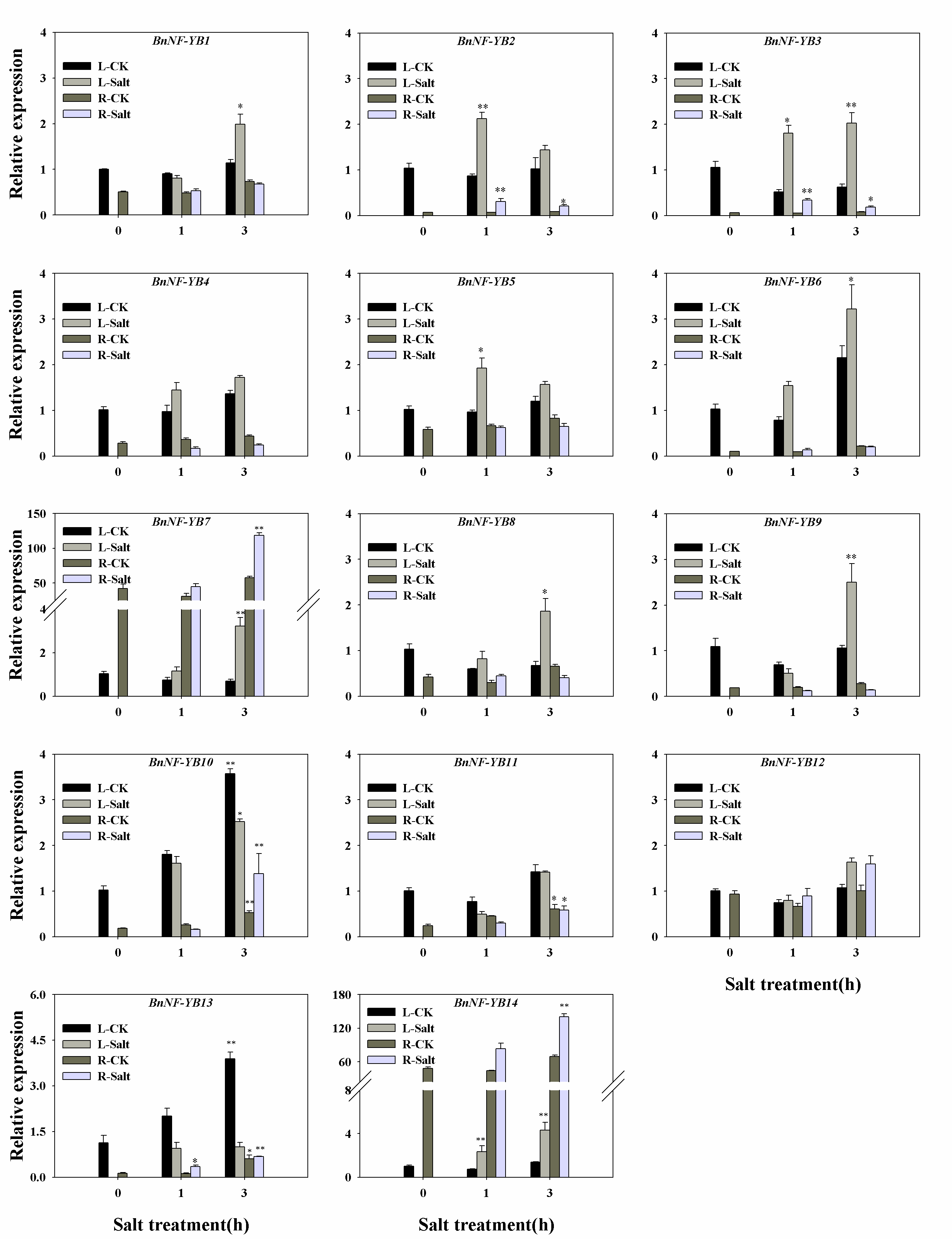


**BnNF-YC Subfamily (C)**


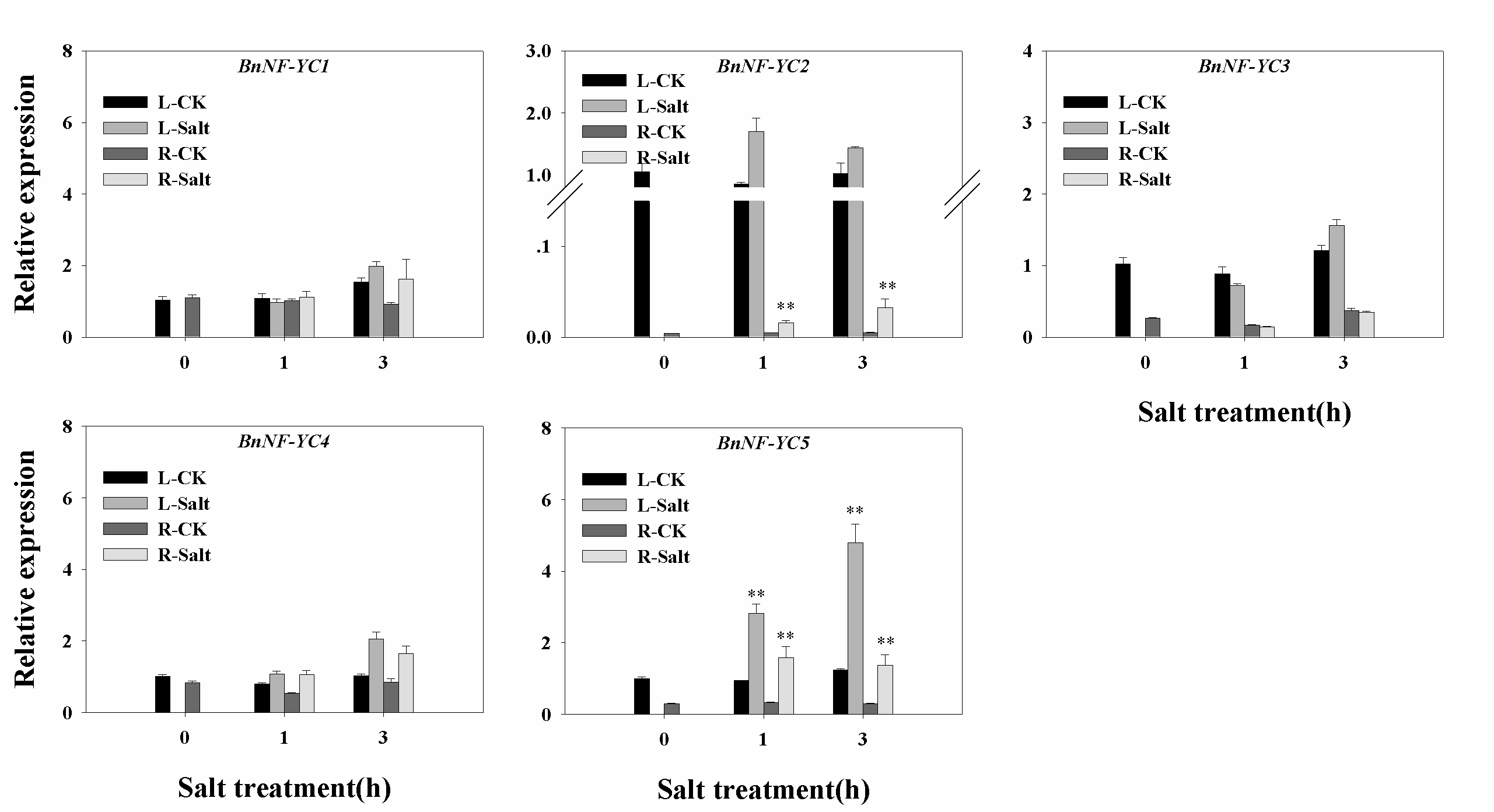


**Supplementary Fig. S1 Expression pattern of *BnNF-Y* genes in the leaves and roots of plants subjected to salinity stress for 1 or 3 h.**
