# Supplementary material for: Multiple NUCLEAR FACTOR Y Transcription Factors Respond to Abiotic Stress in Brassica napus L
Source: PLoS One. 2014 Oct 30;9(10):e111354. doi: 10.1371/journal.pone.0111354 (PMC4214726; doi:10.1371/journal.pone.0111354)
Supplement: Figure S3 — Expression pattern of the BnNF-Y genes after exposure to 100 µM ABA. The expression of BnNF-YA (A), BnNF-YB (B), and BnNF-YC (C) genes in the leaves and roots of plants exposed to 100µM ABA. Transcript levels of each BnNF-Y were first normalized to those of the housekeeping gene 18S and then compared to levels at 0h in the control (untreated) leaves. Expression levels in untreated samples (CK, 0-h leave samples) were arbitrarily set to 1.0. L, leaves; R, roots. CK, no treatment; ABA, ABA treatment. Significant differences between different samples and 0-h samples (same tissue only) are indicated by a single (P<0.05) or double (P<0.01) asterisk, according to Dunnett’s method of one-way ANOVA in SPSS. (DOC) [file pone.0111354.s003.doc]

**BnNF-YA Subfamily (A)**


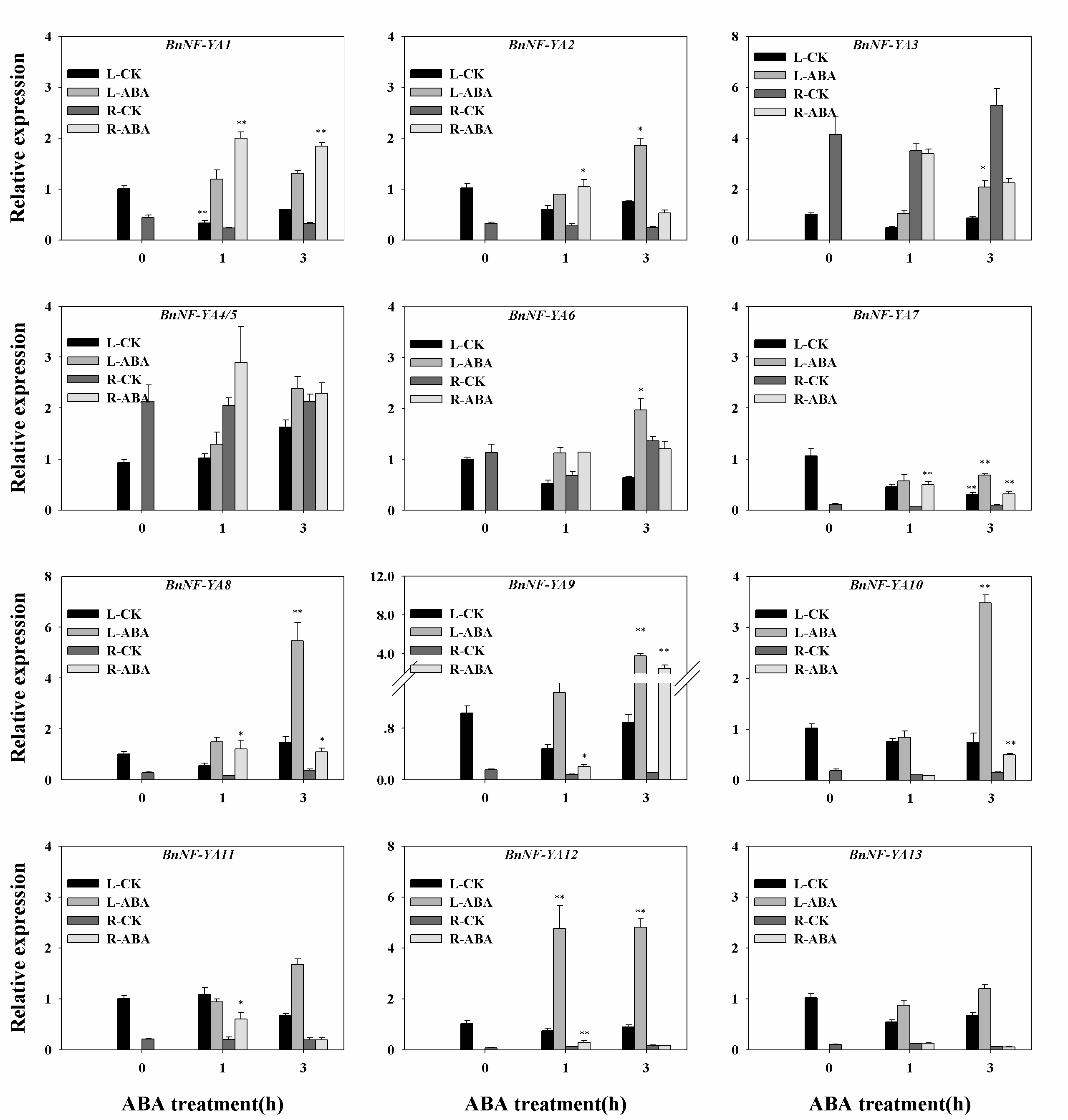


**BnNF-YB Subfamily (B)**


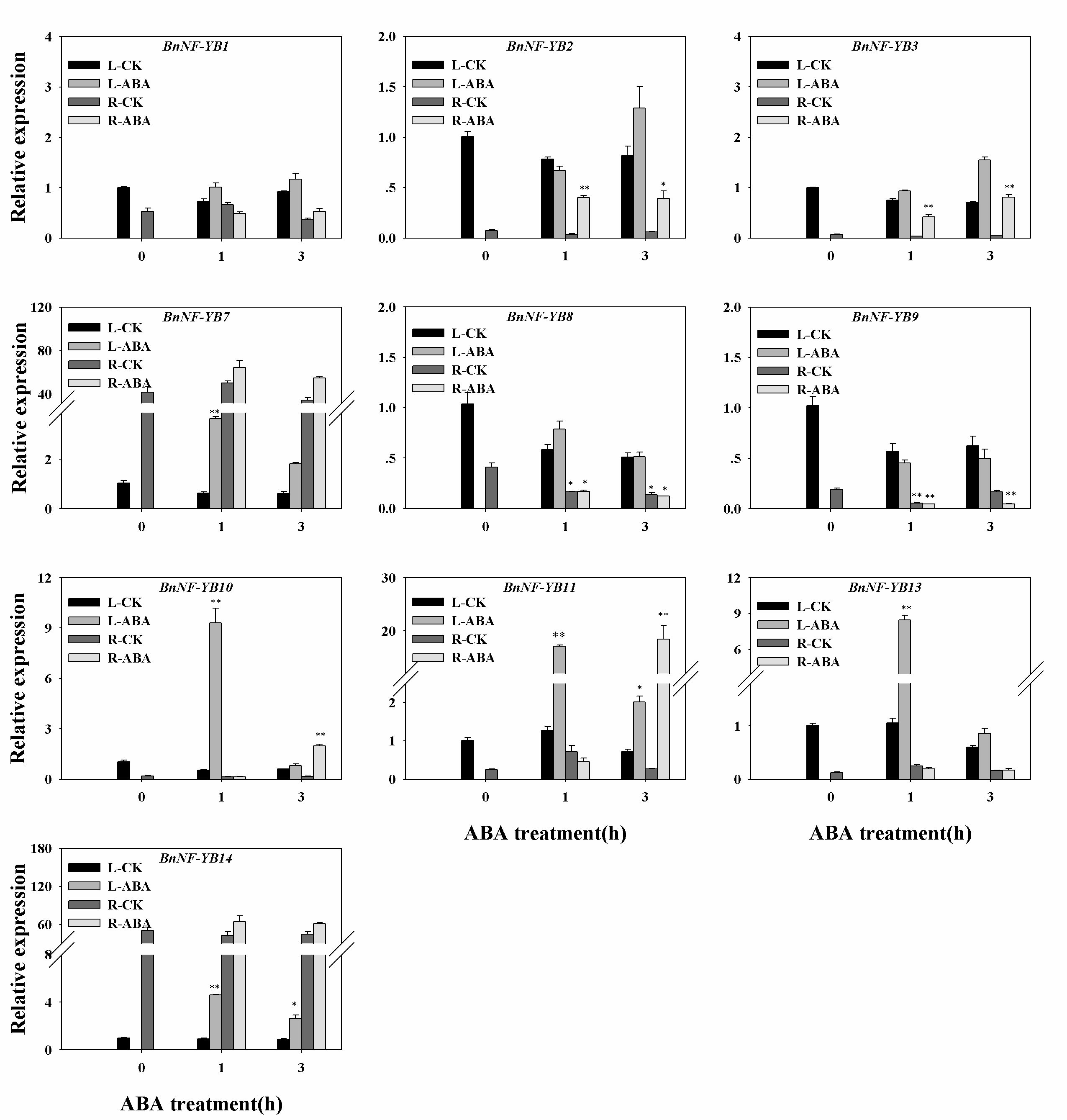


**BnNF-YC Subfamily (C)**


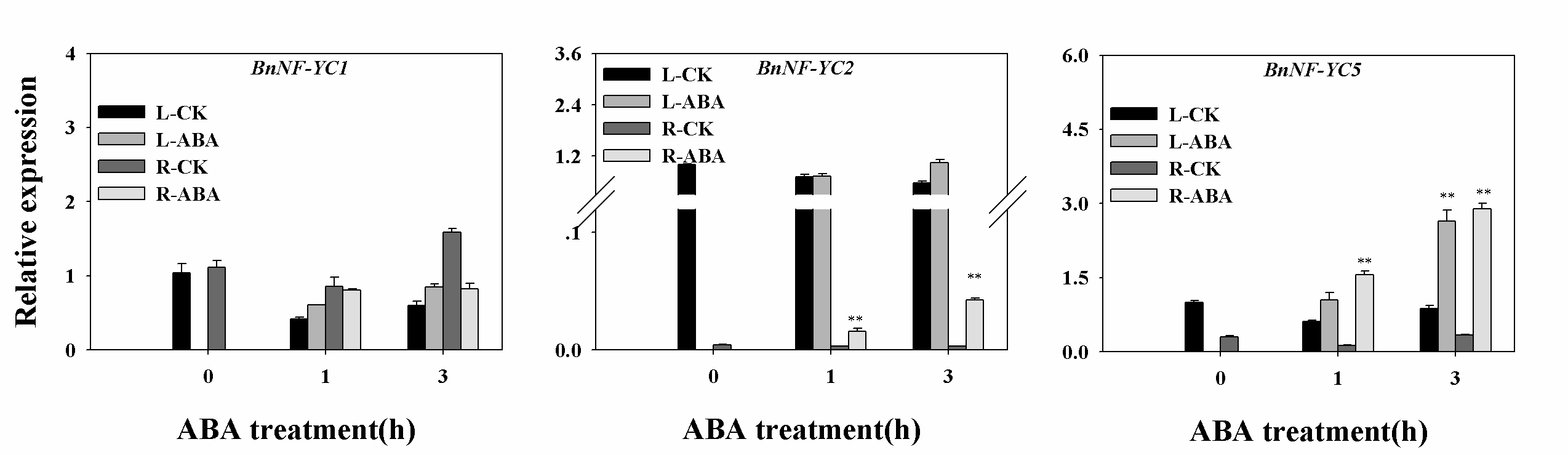


**Supplementary Fig. S3** **Expression pattern of the *BnNF-Y* genes after exposure to 100 μM ABA.** The expression of *BnNF-YA* (A), *BnNF-YB* (B), and *BnNF-YC* (C) genes in the leaves and roots of plants exposed to 100 μM ABA.
