# Supplementary material for: Multiple NUCLEAR FACTOR Y Transcription Factors Respond to Abiotic Stress in Brassica napus L
Source: PLoS One. 2014 Oct 30;9(10):e111354. doi: 10.1371/journal.pone.0111354 (PMC4214726; doi:10.1371/journal.pone.0111354)
Supplement: Figure S4 — Semi-quantitative RT-PCR analysis of BnNF-Y expression in control leaves and roots. RT-PCR analysis was performed on untreated leaf (L) and root (R) tissue samples. The 18S housekeeping gene and BnNF-Ys were amplified for 28 cycles and 35 cycles, respectively. (DOC) [file pone.0111354.s004.doc]

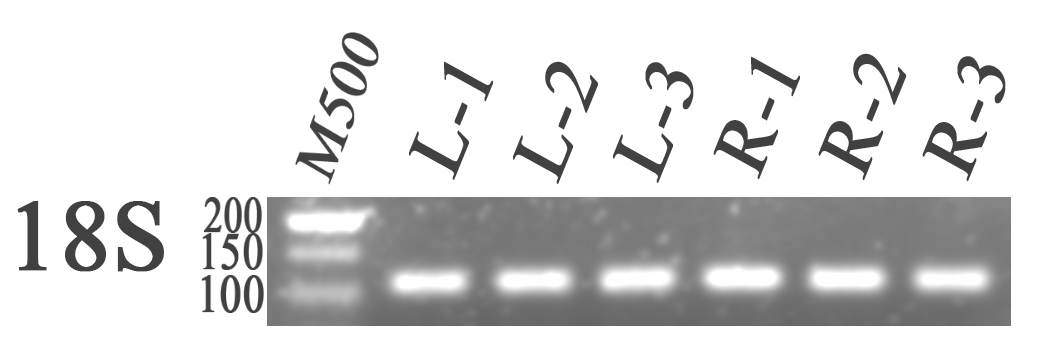


**28 cycles**


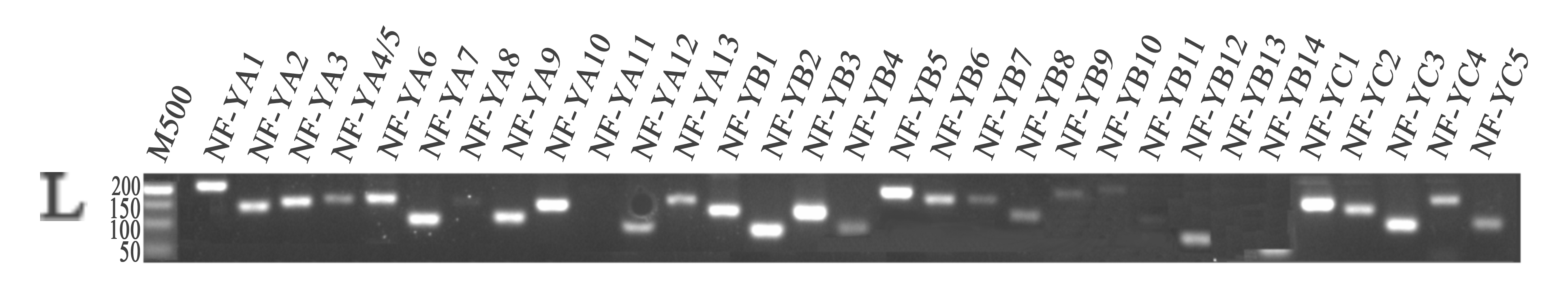


**35 cycles**


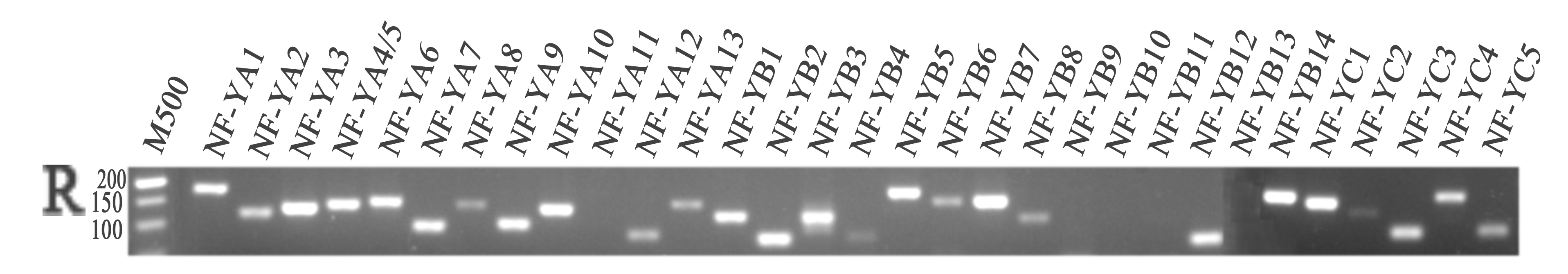


**35 cycles**

**Supplementary Fig. S4** **Semi-quantitative RT-PCR analysis of BnNF-Y expression in control leaves and roots.**
