# Supplementary material for: Multiple NUCLEAR FACTOR Y Transcription Factors Respond to Abiotic Stress in Brassica napus L
Source: PLoS One. 2014 Oct 30;9(10):e111354. doi: 10.1371/journal.pone.0111354 (PMC4214726; doi:10.1371/journal.pone.0111354)
Supplement: Figure S5 — Diagrammatic representation of NF-Y promoter regions. Arabidopsis NF-YA (A), NF-YB (B), and NF-YC (C) promoter regions. Symbols above the top line indicate elements in the forward orientation, and those below the bottom line are in the reverse orientation. MYC(CANNTG) sequences are between the double lines, to indicate their palindromic nature. Numbers are the distance in nucleotides between the sequences and ATG start codon. (DOC) [file pone.0111354.s005.doc]

***Arabidopsis* NF-YA promoter (A)**

***Arabidopsis* NF-YB Subfamily (B)**

***Arabidopsis* NF-YC Subfamily (C)**

**Supplementary Fig. S5 Diagrammatic representation of NF-Y promoter regions.** *Arabidopsis* NF-YA (A), NF-YB (B), and NF-YC (C) promoter regions.
