# Supplementary material for: Multiple NUCLEAR FACTOR Y Transcription Factors Respond to Abiotic Stress in Brassica napus L
Source: PLoS One. 2014 Oct 30;9(10):e111354. doi: 10.1371/journal.pone.0111354 (PMC4214726; doi:10.1371/journal.pone.0111354)
Supplement: Table S1 — Primers for BnNF-Y promoters. F indicates the PCR forward primer and R indicates the PCR reverse primer. Particularly, SP1, SP2, SP3 are three specific primers for Genome Walking. (DOC) [file pone.0111354.s006.doc]

Supplementary Table S1 Primers for *BnNF-Y* promoters. F indicates the PCR forward primer and R indicates the PCR reverse primer. Particularly, SP1, SP2, SP3 are three specific primers for Genome Walking.

| Gene name | Primers (5’to3’) | |
| --- | --- | --- |
| BnNF-YA1 | SP1: TTGAGAATGATGGCGCTTAGCAAC  SP3: GAAGCACAGAAGGTCGCTCTTGTG | SP2: CACAGCTTGTGAATCCTTCCAAGC |
| BnNF-YA2 | F: GAACCGAGAAACATGACC | R: ATCAACACGCATCCCAAG |
| BnNF-YA3 | F: TGACAACCCTAGCACTAC | R: CACACAAGATGACTCCCT |
| BnNF-YA4/5 | F: AACACGACACACACTCTCTG | R: CTGACCTGACTTTGTTGA |
| BnNF-YA6 | SP1: CTTCGCATTGACAAAGACTGGC  SP3: TGCAAATCCCTTATGCTGGGTT | SP2: GAAATTAGCCTGCGTTGGAGCA |
| BnNF-YA7 | F: GATGTTACTACCTGTTAG | R: TTTGGGAATCTGGACGTT |
| BnNF-YA8 | F: TACAAGTGTAGCTGCATATAAG | R: AATCTTTGCTTTGGAACACTTGCAT |
| BnNF-YA9 | F: ACCAAAACTTGAAAATGA | R: TGTTTGAATTTGTTGGCACTTGCAT |
| BnNF-YA10 | F: CTTATCTTAGCTTTCCAT | R: CTTAACCAAGAGAGCACC |
| BnNF-YA11 | F: AGGGAATCCAATGAAGTGACTCGTG | R: TCCCACTTTCAGATTTTGACTGCAT |
| BnNF-YA12 | F: AGGGAATCCAATGAAGTGAC | R: TGCATGTCTTCAGTTTCCAT |
| BnNF-YA13 | F: TATTTGCGTTTTGGAAGC | R: CAATTATGATCCCACTTC |
| BnNF-YB1 | F: CCATTGGTCGGTGTAGC | R: CATCTTTCCCGATCTTGC |
| BnNF-YB2 | F: ATCCATAGGGCTTAGG | R: GGAGACTGTCCGTTTT |
| BnNF-YB3 | F: TAAGGGATTTGAGAAGTA | R: TAAACTCGGAGACGCACT |
| BnNF-YB4 | F: TTTTTACTGATAAGACTCAACTTTC | R: TTGAAAGTTGAGTCTTATCAGTAAA |
| BnNF-YB5 | F:TGGGTTGAGTTTCTTAGTTCGCT | R: GTGACCGCCTGAATCGTTATC |
| BnNF-YB6 | F: CTTTGAAACTCATTGATGTTGTCCT | R: TCGGGAGAAACCTATCCTGCT |
| BnNF-YB7 | F: AGAAGAACTAAAGACGCA | R: TTCCACCACTAAACCATC |
| BnNF-YB8 | F:CCTCACTATTCCCCTTCC | R: TAGTCTGGTTGGGCTCTG |
| BnNF-YB9 | F: CTTTCACGCCGCCCAT | R: AGAACTTGCCGTAGCCAT |
| BnNF-YB10 | F: TTTTGTCTTGTCGTGGTG | R: ATTTGTTTCGGCTACTCC |
| BnNF-YB11 | F: TTGTCAATCGTAGTG | R: TTCCTCTGGGCTCTCCTC |
| BnNF-YB12 | F: CCAAGTTATGCCACG | R: TTTGATTACCCGAGTTAG |
| BnNF-YB13 | F: GAGCAGTGGAGGAAAGCG | R: CTTCCACCGCCTCCTACT |
| BnNF-YC1 | F:TATACAAATGACTAATCC | R:ATAGTGTTTGTATGAGAA |
| BnNF-YC2 | F:AATGAAGTGGGTGTTTGA | R:GAAGTCTGTGGTCTGCTC |
| BnNF-YC5 | F:TTGGGATACACCACACTG | R:CAAACACAACGGGAGCCT |
