# Supplementary material for: Multiple NUCLEAR FACTOR Y Transcription Factors Respond to Abiotic Stress in Brassica napus L
Source: PLoS One. 2014 Oct 30;9(10):e111354. doi: 10.1371/journal.pone.0111354 (PMC4214726; doi:10.1371/journal.pone.0111354)
Supplement: Table S2 — Relative water content (RWC) of drought-stressed plants. Plants were treated with 10%, 15% and 20% (w/v) PEG6000 solution for 24 h. (DOC) [file pone.0111354.s007.doc]

**Supplementary Table 2 Relative water content (RWC) of drought-stressed plants.** Plants were treated with 10%, 15% and 20% (w/v) PEG6000 solution for 24h.

| **Concentration** | **10%PEG** | **15%PEG** | **20%PEG** |
| --- | --- | --- | --- |
| **RWC** | **0.82±0.05** | **0.72±0.02** | **0.54±0.02** |
